# Essential Oils Improve the Survival of Gnotobiotic Brine Shrimp (*Artemia franciscana*) Challenged With *Vibrio campbellii*

**DOI:** 10.3389/fimmu.2021.693932

**Published:** 2021-10-20

**Authors:** Xiaoting Zheng, Biao Han, Vikash Kumar, Adam F. Feyaerts, Patrick Van Dijck, Peter Bossier

**Affiliations:** ^1^ Laboratory of Aquaculture & Artemia Reference Center, Department of Animal Science and Aquatic Ecology, Faculty of Bioscience Engineering, Ghent University, Ghent, Belgium; ^2^ Key Laboratory of South China Sea Fishery Resources Exploitation & Utilization, Ministry of Agriculture and Rural Affairs, South China Sea Fisheries Research Institute, Chinese Academy of Fishery Sciences, Guangzhou, China; ^3^ Key Laboratory for Healthy and Safe Aquaculture, Institute of Modern Aquaculture Science and Engineering, School of Life Sciences, South China Normal University, Guangzhou, China; ^4^ Aquatic Environmental Biotechnology & Nanotechnology (AEBN), ICAR-Central Inland Fisheries Research Institute, Kolkata, India; ^5^ Vlaam Instituut voor Biotechnologie, Katholieke Univeriteit (VIB-KU) Leuven Center for Microbiology, Leuven, Belgium; ^6^ Laboratory of Molecular Cell Biology, Institute of Botany and Microbiology, Katholieke Univeriteit (KU) Leuven, Leuven, Belgium

**Keywords:** essential oil, *Vibrio campbellii*, *Artemia franciscana*, survival, immune gene, virulence factor

## Abstract

The halophilic aquatic bacterium *Vibrio campbellii* is an important aquatic pathogen, capable of causing vibriosis in shrimp and fish resulting in significant economic losses. In a previous work, essential oils (EOs) extracts from *Melaleuca alternifolia*, *Litsea citrata*, and *Eucalyptus citriodora* were found to inhibit the growth of *V. campbellii in vitro*. This study aimed to determine *in vivo* EOs’ potential protective effect towards gnotobiotic brine shrimp *Artemia franciscana*, challenged with *V. campbellii*. The study showed that brine shrimp larvae supplemented with EOs of *M. alternifolia* (0.0008%) and *L. citrata* (0.002%) displayed significantly increased survival against *V. campbellii*. The results indicated that supplementation of these EOs increased the expression of immune-related genes (either in the presence or absence of the pathogen), probably contributing to enhanced protection. Furthermore, *in vitro* studies indicated that some EOs modulated the expression of virulence factors including swimming motility, biofilm formation, and gelatinase and lipase activity, while flow cytometry data and regrowth assay indicated that these EOs do not exhibit antimicrobial activity as *V. campbellii* grew at the tested concentrations [*M. alternifolia* (0.0008%) and *L. citrata* (0.002%)]. Our findings suggest that EOs extracted from *M. alternifolia* and *L. citrata*, can modulate virulence factor production and immunological responses and might hence become part of an intervention strategy to control vibriosis in a fish or shrimp aquaculture setting, a hypothesis that needs to be validated in the future.

## Introduction

The Gram-negative motile marine bacterium *Vibrio campbellii* is one of the most important pathogens in shrimp or fish culture, resulting in serious economic losses in aquaculture (1). The pathogenicity of *V. campbellii* is determined by its ability of biofilm formation, swimming motility, and the production of various degradative enzymes, such as hemolysins, gelatinases, lipases, and phospholipases (2). In many cases, *Vibrio* is an opportunistic pathogen. They cause disease only when the host organism is immune suppressed or otherwise physiologically stressed (3). Therefore, to prevent *V. campbellii*–based vibriosis in aquatic animals, an approach that focuses on understanding the host immune response of any potential mitigation strategy is warranted.

Traditionally, the non-judicious use or overuse of antibiotics and disinfectants applied to cure infections caused by *Vibrio* had limited success, resulting in the emergence of bacterial resistance (4). Because of such concerns, there is an urgent need for the development of plant-derived compounds or natural products that protect aquatic animals and enhance the immune reactivity towards *V. campbellii* infection. However, a major challenge in studying the biological activity of natural compounds *in vivo* is the difficulty to either eliminate or extricate the influence of the microbial communities that occur in the natural environment. Besides, in germ-associated conditions, natural components are not only metabolized by the microbial communities but also influence the physiology of the host-related microbes (5). Consequently, it is paramount to select an appropriate animal model system that permits better delineation of the biological effects of the natural components.

The brine shrimp *Artemia franciscana* is an aquatic invertebrate that can be reared under gnotobiotic conditions (a germ-free system that allows for full control over the host-associated microbial communities). This model allows for distinguishing the direct effect on the host (by pre-exposing axenic brine shrimp larvae to a compound of interest for a certain duration) from indirect effects (6).

The International Standard Organization (ISO) defines essential oils as concentrated relatively hydrophobic liquids containing relatively volatile chemical compounds. They can be obtained from different parts of the plant, such as seeds, roots, buds, leaves, flowers, peels, and fruits, by the methods of steam distillation or (cold) pression (7). Given their bactericidal and fungicidal properties, EO application in the aquaculture industry is becoming more and more widespread as an alternative in controlling pathogens. For instance, the EO of *Syzygium aromaticum* inhibits fish systemic bacteria (8). Oregano essential oil can not only act as a growth promoter but also improves resistance to *Aeromonas hydrophila* when supplied to channel catfish *(Ictalurus punctatus)* feed (9). The EOs extracted from the leaves of *Lippia sidoides* and *Mentha piperita* can kill the gill parasites of Nile tilapia (10).

Furthermore, in our previous work, 22 different EOs were screened for their possible anti–*V. campbellii* activity (11). The results demonstrated that EOs of *Melaleuca alternifolia*, *Litsea citrata*, and *Eucalyptus citriodora* were the three best candidates to inhibit the growth of *V. campbellii.* However, whether the selected EOs could modulate the *in vitro* production of *V. campbellii* BB120 virulence factors was not documented. Additionally, the immunomodulatory properties of these EOs that could lead to enhanced protection against pathogenic *V. campbellii* BB120 of the experimental animals have not been verified in this model. Hence, the present study aimed to investigate whether the selected EOs induce an innate immune response in brine shrimp larvae and/or regulate the virulence of pathogenic *V. campbellii in vitro*.

## Material and Methods

### Selected EOs and DMSO

EOs of *Melaleuca alternifolia*, *Litsea citrata*, and *Eucalyptus citriodora* were purchased from Pranarôm International S.A. (Ghislenghien, Belgium). Based on our previous study, the three essential oils mentioned above were selected to use in this study (11). The chemical composition of these three essential oils was characterized (**Supplementary Information 1**). All EOs were kept in the brown sterile glass vial and stored at 4°C. Dimethyl sulfoxide (DMSO) was purchased from VWR International (Leuven, Belgium).

### *Vibrio campbellii* Strain and Growth Conditions

*V. campbellii* wild-type strain ATCC BAA-1116 (BB120), stored in 20% sterile glycerol at −80°C, was used in the challenge assays. This strain was reactivated by streaking from the stock solution onto Luria-Bertani agar plates (Carl Roth, Karlsruhe, Germany) containing 35 g/L of sodium chloride (LB_35_). Subsequently, a pure colony was transferred to and cultured overnight in LB_35_ broth (Carl Roth, Karlsruhe, Germany) by incubation at 28°C with continuous shaking (120 rpm). Bacterial cell densities were measured by spectrophotometry at 600 nm.

### Axenic Hatching of Brine Shrimp Larvae *Artemia franciscana*

Axenic brine shrimp larvae were obtained following the decapsulation and hatching process based on the method described before (12). Briefly, 200 mg of brine shrimp cysts originating from the Great Salt Lake, Utah, USA (EG Type, batch 1871, INVE Aquaculture, Dendermonde, Belgium) were hydrated in 18 ml of filter-sterilized distilled water for 1 h. Then, sterile cysts and larvae were obtained by decapsulation, adding 660 µl NaOH (32%) and 10 ml NaClO (50%). The decapsulation process was stopped after about 2 min by using 14 ml Na_2_S_2_O_3_ at 10 g/L. Filtered aeration (0.22 µm) was provided during the reaction. The aeration was then terminated, and the decapsulated cysts were washed with filtered (0.2 µm) autoclaved artificial seawater (FASW) containing 35 g/L of instant ocean synthetic sea salt (Aquarium Systems, Sarrebourg, France). All manipulations were conducted under a laminar flow hood, and all tools were autoclaved at 121°C for 20 min. Afterwards, the cysts were suspended in a 50 ml falcon tube containing 30 ml FASW and incubated for 28 h on a rotor at 6 rpm at 28°C with constant illumination of approximately 2,000 lux.

### Evaluation of Selected EOs Toxicity in Brine Shrimp Larvae

After 28 h of hatching, the larvae at instar II stage (when they started ingesting particles) were randomly collected (n=20) and transferred to fresh, sterile 40 ml glass tubes containing 10 ml of FASW. The glass tubes with axenic larvae were added with increasing concentrations of essential oil from 0.0005 to 0.005%, in 1% of DMSO and fed once with 10^7^ cells/ml autoclaved LVS3 (a feed for *Artemia*). The control group were supplemented with only 1% of DMSO. Afterwards, the tubes were put to the rotor and kept at 28°C. Survival of the larvae was recorded at 48 h after exposure to EOs. Four replicates were maintained for all treatments, and each experiment was repeated twice to verify the reproducibility. In each experiment, the sterility of the control and treatment groups was checked at the end of the toxicity assay by inoculating 1 ml of rearing water to 9 ml of LB_35_ and incubating the mixture for 2 days at 28°C (13).

### Brine Shrimp Larvae Challenge Assay

The challenge test was performed as described by Yang et al. (12) with some modifications. Twenty larvae at development stage II were collected and transferred to fresh, sterile 40 ml glass tubes containing 10 ml of FASW as described above in the toxicity assay. A suspension of autoclaved LVS3 bacteria in each glass tubes was added as feed at the start of the challenge test at 10^7^ cells/ml in all the treatments. Subsequently, the tubes were supplemented with increasing concentrations of selected EOs from 0.0001 to 0.001% (*M. alternifolia*), 0.003% (*L. citrata*), and 0.0008% (*E. citriodora*), respectively, in 1% of DMSO. The larvae not supplemented with EOs and challenged with *V. campbellii* served as the positive control, while non-supplemented larvae groups without challenge (but with 1% of DMSO supplemented) were used as the negative control. Afterwards, the larvae were challenged with *V. campbellii* at 10^7^ cells/ml. The survival of larvae was scored after 48 h of *V. campbellii* challenge. Four replicates were maintained for all treatments, and the experiment was repeated twice to verify the reproducibility. In the experiment, the sterility of the negative control group was checked at the end of the survival assay by inoculating 1 ml of rearing water to 9 ml of LB_35_ and incubating the mixture for 2 days at 28°C (13). Relative percent of survival (%, RPS) was calculated by equation: = (1 − (% mortality in the EO treated group/% mortality in the control group)) × 100 (14).

### Assay of Immune-Related Genes Expression in the Optimized Dose of EOs by Reverse Transcriptase Real-Time PCR

After 28 h incubation at 28°C, the instar II stage swimming *Artemia* nauplii were collected, counted volumetrically, and transferred to 1 L sterile glass bottles containing 500 ml FASW. The nauplii were fed once with 10^7^ cells/ml of autoclaved LVS3. Afterwards, the nauplii were treated with an optimized dose of selected essential oils (based on the result of the dose-response study) and challenged with *V. campbellii* at 10^7^ cells/ml. Non-challenged larvae groups (in 1% of DMSO) were used as control. Each treatment was carried out in triplicate. Samples containing 25 mg of live larvae were harvested from all treatments and control groups at 6, 12, and 24 h after addition of *V. campbellii*, rinsed in distilled water, immediately frozen in liquid nitrogen, and stored at −80°C.

Total RNA was extracted from control and treatment group larvae samples using the RNeasy Plus Mini Kit (Qiagen, Germany) according to the manufacturer’s instructions. The genomic DNA was eliminated with a gDNA eliminator spin in the kit when isolating the RNA. The quality and quantity of RNA were confirmed by NanoDrop™ 2000 (Thermo Scientific, USA). Then 2 µg of total RNA was used to synthesize the first-strand cDNA using RevertAid H Minus First Strand cDNA Synthesis Kit (Thermo Scientific, USA) with an oligo (dT) primer. The reverse transcription quantitative PCR (RT-qPCR) assay was performed on Step One Plus Real-Time System (Applied Biosystems, USA) using Maxima SYBR Green/ROX qPCR Master Mix (2X) (Thermo Scientific, USA) in a total volume of 20 µl, containing 10 µl of 2X SYBR green master mix, 1 µl of forward and reverse primers (10 nM), 1 µl of template cDNA (10 ng), and 7 µl of nuclease-free water. The thermal cycling consisted of an initial denaturation at 95°C for 10 min followed by 40 cycles of denaturation at 95°C for 15 s and primer annealing and elongation at 60°C for 1 min. Dissociation curve analysis was performed to check for the amplification of untargeted fragments. Data acquisition was performed with the Rotor-Gene Q software version 2.0.2 (Qiagen, Germany). Two internal reference genes, *EF-1* and *GAPDH*, were introduced to normalize the qPCR data. Gene specific primers (designed by cross-exon strategy and the software PRIMER PREMIER version 5.0) were described by previous studies (15) and shown in **Table 1**.

**Table 1:** Specific primers used for reverse transcriptase qPCR.

Gene	Primer sequences (5’-3’)
EF-1	TCGACAAGAGAACCATTGAAAA
	ACGCTCAGCTTTAAGTTTGTCC
GAPDH*	GTTGATGGCAAACTCGTCATA
	CCACCTTCCAAGTGAGCATTA
*hsp70*	CGATAAAGGCCGTCTCTCCA
	CAGCTTCAGGTAACTTGTCCTTG
*sod*	CAATCAGCATTGGGGTTTGTC
	GAATCTCTTCGTTGGTTGTAGGG
*dscam*	TCAAGAGGCTGAAAGAGAAGAAAT
	CAGTAGAAGCAGTGACCCAGAAAT
*lgbp*	CCGTGAAGATCCCAACGAAC
	GGAGGAGGTAATTGGGAGTTTCAAGG
*hmgb*	AGAGGCGGGAAAGGAAGC
	CCCACACCAAGACCAGGTTG
*pxn*	TTGGTGCTGCTGCTTTTCG
	CCCCATCGCTTGTCTTCGT
*tgase 1*	GCAAGGAGCTGGAATGGGT
	TGTTTGGGAGTTAATCGGACTGT
*tgase 2*	TTCTTTACACAGGCATTCCGTC
	GTTACATCAAATCCCAGCTCCA

EF-1, elongation factor 1; GAPDH, glyceraldehyde-3-phosphate dehydrogenase; hsp70, heat shock protein 70; sod, superoxidase dismutase; dscam, down syndrome cell adhesion molecule; lgbp, lipopolysaccharide and β-1,3-glucan-binding protein; hmgb, high mobility group box protein; tgase 1, transglutaminase 1; tgase 2, transglutaminase 2.

*Adapted from Chen et al. (16).

The expression of the target genes was normalized to the endogenous control (Geomean of *EF-1* and *GAPDH*) by calculating ΔC_T_:


$$\Delta {\text{C}}_{\text{T}}={\text{C}}_{\text{T},\text{ target}}-{\text{C}}_{\text{T},\text{ Geomean of }EF-1\text{ and }GAPDH}$$

and expressed relative to the calibrator sample by calculating ΔΔC_T_:


$$\Delta \Delta {\text{C}}_{\text{T}}=\Delta {\text{C}}_{\text{T}}-\Delta {\text{C}}_{\text{T},\text{calibrator}}$$

The sample of unchallenged *Artemia* (1% of DMSO) at 6 h was used as a calibrator. The relative expression was then calculated as


$$\text{Relative expression}=2^{-\Delta \Delta \text{CT}}$$

At each time point, the relative gene expression in the unchallenged control group was set at 1.0, and the gene expression of the remaining groups was normalized accordingly. If the fold change was significant and higher than 2, the EO was considered to have an immunostimulatory effect.

### Effect of Selected Essential Oils on Cell Viability and Growth Performance of *V. campbellii*

To determine the effect of selected essential oils on the viability of bacterial cells, the proportion of live or dead cells in the bacterial culture was investigated by flow cytometry and regrowth test. For that, the concentration of overnight *V. campbellii* culture was adjusted to 10^8^ cells/ml and was subsequently diluted with FASW to 10^6^ cells/ml. Then, the EOs of *M. alternifolia* (at the concentrations of 0.0005, 0.0008, 0.001, and 0.01%), *L. citrata* (at the concentrations of 0.001, 0.002, 0.003, and 0.01%), and *E. citriodora* (at the concentrations of 0.0003, 0.0005, 0.0008, and 0.01%) were dissolved in 1% DMSO and added to 1 ml of the acquired bacterial culture in Eppendorf tubes. The tubes containing only 1% DMSO with bacterial culture served as the negative control. Afterwards, all Eppendorf tubes were incubated at 28°C for 1 h on the rotor (6 rpm).

Later, 100 µl of the bacterial culture was placed into each well of the 96-well black microtiter plate with the flat bottom (SPL Life Sciences) together with 5 µl Thiazole Orange (17 mM) and 5 µl Propidium Iodide (0.15 mM). The mixture of 100 µl of FASW and dyes was used as the blank. The experiment was conducted in triplicate, corresponding to three wells of the plate. The plate was then covered by the lid for 10 min for the staining reaction. The amount of live and dead bacterial cells was then determined by the CytoFLEX instrument (Beckman Coulter, USA).

For the regrowth assay, 10 µl of the bacterial culture was added to 990 µl of LB_35_ broth. Then, 200 µl volume of this suspension was put in 96-well transparent plate with the flat bottom (VWR International) at 28°C with shaking for 48 h, and the cell density at 600 nm was monitored every hour using a Tecan Infinite 200 microplate reader (Tecan, Mechelen, Belgium) after resuspending the cells. Each concentration of EO had four replicates, and the growth curve was determined for three independent cultures.

### Swimming Motility Assay

The swimming motility assay was performed on soft agar (LB_35_ plates containing 0.2% agar) as described previously (12) with some modification. The optimized doses of essential oils (based on the dose-response result) dissolved in 1% DMSO were added to the autoclaved agar, when the agar was cooled down to 40°C. Each plate was closed with a lid immediately after pouring the medium to maintain equal moisture in plates by preventing evaporation. *V. campbellii* was grown overnight in LB_35_ broth, and 2 µl aliquots (OD_600_ = 0.1) were spotted in the center of the soft agar plates. Plates were incubated at 28°C for 24 h, after which the diameters of the motility halos were measured. All assays were done with freshly prepared media in four replicates.

### Biofilm Formation Assay

Biofilm formation assay was quantified by crystal violet staining, as described previously (12). In brief, *V. campbellii* was grown overnight and diluted to an OD_600_ of 0.1 in marine broth with or without essential oils, and 200 µl aliquots of these suspensions were pipetted into the wells of a 96-well plate. Then, the bacteria suspensions were incubated without agitation for 24 h at 28°C. After that, the cultures were removed, and the wells were washed three times with 300 µl sterile physiological saline to remove all non-adherent bacteria. The remaining attached bacteria were fixed with 200 µl/well of 99% methanol for 2 h, after which the methanol was removed and plates were air-dried overnight. Then, biofilms were stained for 20 min with 200 µl/well of a 0.1% crystal violet solution (Pro-lab Diagnostics, Richmond Hill, ON, Canada). Excess stain was rinsed off by placing the plate under running tap water, and washing was continued until the washings were free of the stain. After the plates were air dried, the dye bound to the adherent cells was resolubilized with 200 µl/well of 95% ethanol, and absorbance was measured at 570 nm with a Multi-reader (Infinite M200, TECAN, Austria). Five replicates of two independent bacteria cultures were maintained for all treatments in this test, and the experiment was repeated twice to verify the reproducibility.

### Lytic Enzyme Activity Assays

All assays were conducted according to Natrah et al. (17). For each assay, an overnight culture of *V. campbellii* was diluted to an OD_600_ of 0.5, and 2 µl of the diluted culture was spotted in the middle of the test plates. The optimized doses of essential oils dissolved in 1% DMSO were added to the autoclaved agar, when the agar was cooled down to 40°C. All assays were done in quintuplicate. Similarly, the lipase and phospholipase activities were assessed on marine agar plates supplemented with 1% Tween 80 (Sigma-Aldrich) and 1% egg yolk emulsion (Sigma-Aldrich), respectively. The development of opalescent zones around the colonies was observed, and the diameters of the zones were measured after 2–4 days of incubation at 28°C. Gelatinase assay plates were prepared by mixing 0.5% gelatin (Sigma-Aldrich) into the agar. After incubation for 2 days, saturated ammonium sulfate (80%) in distilled water was poured over the plates, and after 2 min, the diameters of the clearing zones around the colonies were measured. Hemolytic assay plates were prepared by supplementing Marine Agar with 5% defibrinated sheep blood (Oxoid), and clearing zones were measured after 2 days of incubation.

### Statistical Analyses

Data were expressed as mean ± SEM. The *Artemia* survival data were arcsin transformed to satisfy normal distribution and homoscedasticity requirements as necessary. Lethal concentration in media (LC_50_) was determined by interpolation to a 4PL-Sigmoidal standard curve. Log transformed gene expression data were shown. For each gene, the expression in control was set at 1, and the expression in different treatments was normalized accordingly using the 2^−ΔΔCT^ method. In the interaction studies, statistical analysis was performed using two-way ANOVA followed by Tukey test. Unless stated otherwise, all other data were compared with one-way ANOVA, followed by Tukey’s *post hoc* test. Statistical analysis was performed using the IBM statistical software SPSS (version 22.0, IBM Corp., Armonk, New York, USA), and the results were shown using GraphPad Prism software (version 7, GraphPad Software, Inc., San Diego, CA, USA) and included all biological repeats. The significance level was set at *P* < 0.05.

## Results

### *In Vivo* Toxicity (Lethal Concentration, LC_50-48h_) of EOs

**Figure 1:**
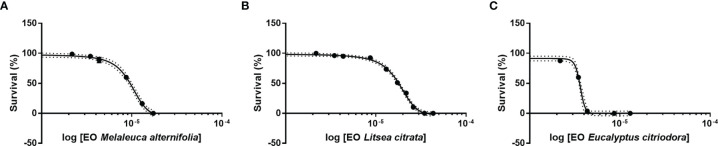
Toxicity of three essential oils (*Melaleuca alternifolia, Litsea citrata,* and *Eucalyptus citriodora*) on *Artemia franciscana*. Survival was recorded 48 h after EOs treatment. **(A)** *Melaleuca alternifolia,* **(B)** *Litsea citrata,* **(C)** *Eucalyptus citriodora*. X axis represented log transformed concentrations data. The error bars represented the standard error of four replicates. Dotted line on charts represents 95% confidence intervals.

Firstly, it was investigated whether the EOs have cytotoxic effects on axenic brine shrimp larvae. Therefore, the toxicity of three EOs was determined for *Artemia* as LC_50-48h_ (**Figure 1**): (i) EO of *M. alternifolia* (2.2492×10^−3%^) with a 95% confidence interval (CI) ranging from 2.0977×10^−3^ to 2.4016×10^−3^%, R^2^ = 0.983), (ii) EO of *L. citrata* (4.1563×10^−3^%, 95% CI = 3.9812×10^−3^ to 4.3313×10^−3^%, R^2^ = 0.987), and (iii) EO of *E. citriodora* (0.8349×10^−3^%, 95% CI from 0.813×10^−3^ to 0.857×10^−3^%, R^2^ = 0.9866). According to **Figure 1**, these results suggested that the exposure with EO *M. alternifolia*, *L. citrata*, and *E. citriodora*, at concentrations with no more than 0.001, 0.002, and 0.0005%, respectively, are non-toxic to brine shrimp larvae and considered as safe concentrations to *Artemia*, with the survival of *Artemia* is > 90%.

### EOs Protect Brine Shrimp Larvae Against Subsequent *V. campbellii* Challenge

**Figure 2:**
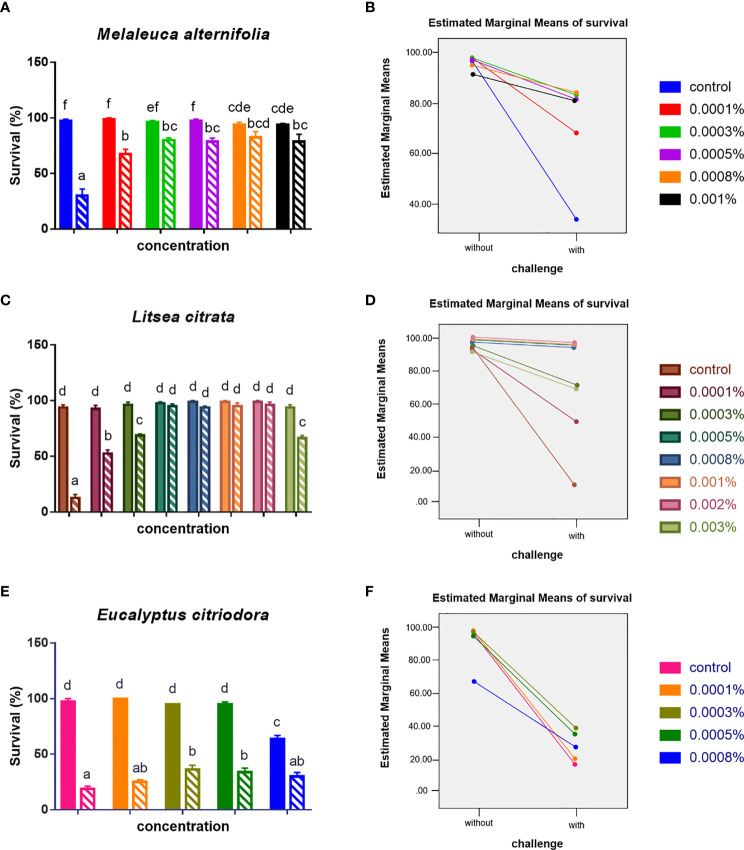
Survival (mean ± SEM) of brine shrimp larvae 48 h after challenge with *Vibrio campbellii* BB120. **(A)** Treated with EO of *Melaleuca alternifolia*, **(C)** treated with EO of *Litsea citrata*, and **(E)** treated with EO of *Eucalyptus citriodora*. **(B, D, F)** showed the interaction plots between doses of each EO and with/without *V. campbellii* challenge, respectively, according to the output from two-way ANOVA. All treatments were carried out in four replicates. The bars without pattern were unchallenged groups; the opposite was challenged groups. Different letters indicated significant difference (*P* < 0.05). Control: no EO added but consisted of 1% of DMSO.

Secondly, it was determined whether the EOs at safe concentrations (EO *M. alternifolia*: 0.0001, 0.0003, 0.0005, 0.0008, and 0.001%; EO *L. citrata*: 0.0001, 0.0003, 0.0005, 0.0008, 0.001, 0.002, and 0.003%; EO *E. citriodora*: 0.0001, 0.0003, 0.0005, and 0.0008%) could protect *Artemia* nauplii against a *V. campbellii* challenge. Furthermore, the effect of two parameters (different concentrations, with/without a *V. campbellii* challenge) and their interaction on the survival of brine shrimp were analyzed. As shown in **Figure 2** and **Tables 2**, **3**, brine shrimp larvae that were treated with EOs at safe doses displayed a significant increase in their survival compared to the control when challenged with *V. campbellii*. As shown previously, the *Vibrio* challenge reduced the survival of brine shrimp larvae. A dose-dependent increase in the survival rate was recorded upon treatment with the EOs. In addition, a significant synergistic interaction between the two experimental factors (namely, EO at different doses and *Vibrio* challenge) was observed, indicating that the EOs protect against the *Vibrio*. The highest relative percent survival was observed in the *Vibrio*-exposed groups treated with *L. citrata* with concentrations of 0.0003–0.002%. However, with further increases in EO concentrations, the survival tended to decrease, probably a result of EO-related toxicity.

**Table 2:** A summary table with EOs’ lethal concentrations to 50% (LC50) value, 10% (LC10) value, and the EOs’ concentration of relative precent of survival 50% (RPS 50) and 90% (RPS 90) of *Artemia*.

Essential oil of plant species	LC 50 (%)	LC 10 (%)	RPS 50 (%)	RPS 90 (%)
***Melaleuca alternifolia***	0.002	0.001	<0.0001	N
***Litsea citrata***	0.004	0.002	<0.0001	0.0003–0.002
***Eucalyptus citriodora***	0.0008	0.0005	N	N

Relative percent of survival (%, RPS) was calculated by equation: = (1 − (% mortality in the EO treated group/% mortality in the control group)) × 100 (14).

N, non-observed.

**Table 3:** Two-way ANOVA showed main and interaction effects of indicated concentrations of essential oils and with or without *Vibrio campbellii* challenge on the survival of brine shrimp larvae.

Essential oil of plant species	Factors	*P* value	*P* value summary
*Melaleuca alternifolia*	concentrations	<0.0001	****
	challenge	<0.0001	****
	concentrations × challenge	<0.0001	****
*Litsea citrata*	concentrations	<0.0001	****
	challenge	<0.0001	****
	concentrations × challenge	<0.0001	****
*Eucalyptus citriodora*	concentrations	<0.0001	****
	challenge	<0.0001	****
	concentrations × challenge	<0.0001	****

The larvae were treated with essential oils at indicated concentrations either alone or challenged with or without V. campbellii at 10^7^ cells/ml of rearing water. Data represent the mean of four replicates. (Two-way ANOVA; ****P < 0.0001).

### EO-Generated Immune Gene Expression Mediates *In Vivo* Protective Response in Brine Shrimp Larvae

Since the survival of *Vibrio*-challenged brine shrimp larvae treated with EOs at an optimized dose was significantly increased, we further investigated whether it was due to the stimulation of immune-related genes expression *in vivo*, either in the absence or presence of *Vibrio* challenge To address this, germ-free *Artemia* were treated with the EOs of *M. alternifolia*, *L. citrata*, and *E. citriodora* at a concentrations of 0.0008, 0.002, and 0.0005%, respectively, and analyzed for immune-related genes expression. The main function of the eight selected genes and their possible relationship with immunity in crustaceans are described in the supplementary information (**Supplementary Information 2**).

**Figure 3:**
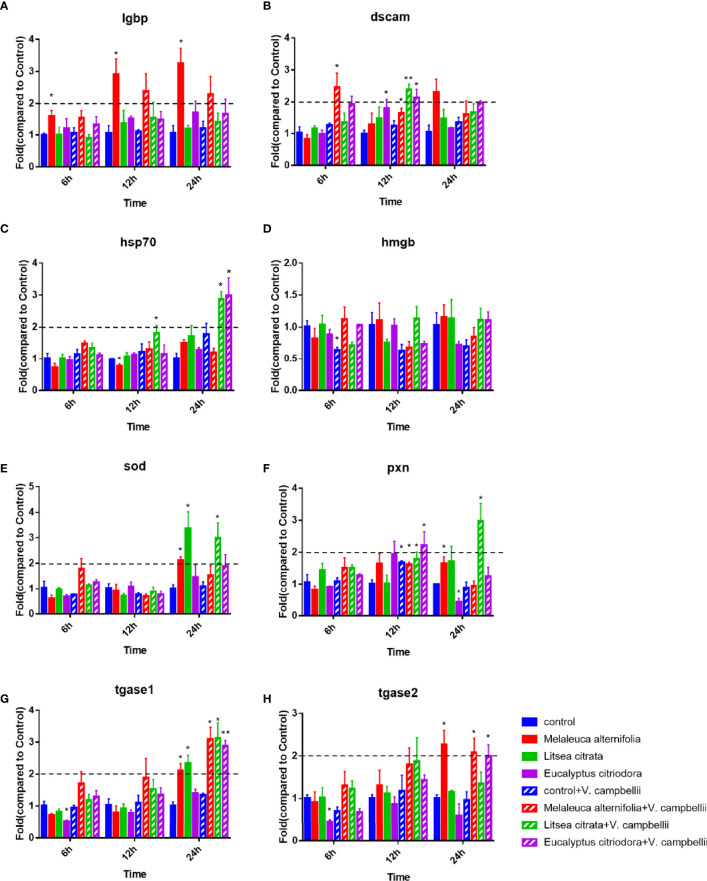
Relative expression of *lgbp* **(A)**, *dscam* **(B)**, *hsp* 70 **(C)** and *hmgb* **(D)**, *sod* **(E)**, *pxn* **(F)**, *tgase 1* **(G)**, and *tgase 2* **(H)** genes in *Artemia* larvae. The unchallenged *Artemia* larvae served as control. For each gene, the expression in control was set at 1, and the expression in different treatments was normalized accordingly using the 2^−ΔΔCT^ method. The geomean of the EF-1 and GAPDH was used as internal control. Data were average ± standard error of three replicates. Asterisks indicated a significant difference when compared to control (independent samples t-test; *P < 0.032; **P < 0.0021). EF-1, elongation factor 1; GAPDH, glyceraldehyde-3-phosphate dehydrogenase; *lgbp*, lipopolysaccharide and β-1,3-glucan-binding protein; *dscam*, down syndrome cell adhesion molecule; *hsp 70*, heat shock protein 70; *hmgb*, high mobility group box protein; *sod*, superoxidase dismutase; *pxn*, peroxinectin; *tgase 1*, transglutaminase 1; *tgase 2*, transglutaminase 2.

If the fold change (in one of the treatments) was higher than 2 and significant, the EO was considered to have an immuno-stimulatory effect. **Figure 3** showed that the expression level of *lgbp* is over two-fold higher at 12 and 24 h in the EO *M. alternifolia* group after the challenge. The *dscam* gene expression level was two-fold higher in the EO *M. alternifolia* group at 6 h, and in the EO *L. citrata* and *E. citriodora* groups at 12 h post challenge. The EO *L. citrata* group challenged with *V. campbellii* exhibited two-fold increase in the *hsp 70*, *sod*, and *pxn* gene expression level at 24 h time point. At 24 h after challenge, the *tgase 1* gene expression in all the EO groups was threefold higher than the control group.

**Figure 4:**
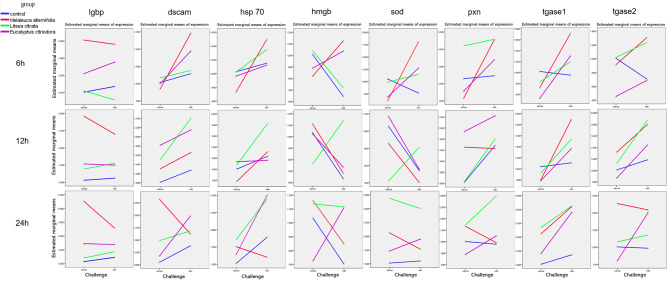
The interaction plots between relative expression of *lgbp*, *dscam*, *hsp* 70, *hmgb*, *sod*, *pxn*, *tgase 1*, and *tgase 2* genes in *Artemia* larvae treated with EO of *Melaleuca alternifolia*, *Litsea citrata*, *Eucalyptus citriodora*, and the control group when challenged with/without *Vibrio campbellii* BB120, respectively, according to the output from two-way ANOVA. Control group: no EO added but consisted of 1% of DMSO.

In addition, **Figure 4** and **Table 4** showed a synergistic interaction of EO (*M. alternifolia*), and the challenge was observed at 6 h in the *dscam* and *hsp 70* gene expression. However, an antagonistic interaction of EO (*M. alternifolia*) and the challenge was observed at 6 h in the *hmgb*, *sod*, and *tagse1* gene expression. There was a significant antagonistic interaction between EO (*E. citriodora*) and the challenge at 6 h in the *hmgb*, *sod*, *tgase1*, and *tgase2* gene expression, and at 24 h in the *hmgb*, *pxn*, and *tgase2* gene expression. In addition, there were no interaction effects between *L. citrata* and challenge in the gene expression.

Table 4Two-way ANOVA showing main and interaction effects of essential oils and with/without *Vibrio campbellii* challenge on the immune gene expressions of brine shrimp larvae at three different time points (6, 12, and 24 h).

**Table:** 

A					
Time point	Gene	Factors	*P* value	*P* value summary	Interaction
6h	*lgbp*	EO (*Melaleuca alternifolia*)	0.0105	*	-
		challenge	0.9619	ns	
		Interaction	0.7353	ns	
	*dscam*	EO (*M. alternifolia*)	0.0842	ns	synergistic
		challenge	0.0055	**	
		Interaction	0.0253	*	
	*hsp 70*	EO (*M. alternifolia*)	0.8680	ns	synergistic
		challenge	0.0104	*	
		Interaction	0.0434	*	
	hmgb	EO (*M. alternifolia*)	0.3109	ns	antagonistic
		challenge	0.8372	ns	
		Interaction	0.0374	*	
	sod	EO (*M. alternifolia*)	0.2526	ns	antagonistic
		challenge	0.0965	ns	
		Interaction	0.0155	*	
	pxn	EO (*M. alternifolia*)	0.6615	ns	-
		challenge	0.1316	ns	
		Interaction	0.1670	ns	
	tgase1	EO (*M. alternifolia*)	0.2742	ns	antagonistic
		challenge	0.0490	*	
		Interaction	0.0291	*	
	tgase2	EO (*M. alternifolia*)	0.2633	ns	-
		challenge	0.8349	ns	
		Interaction	0.1402	ns	
12h	*lgbp*	EO (*M. alternifolia*)	0.0033	**	-
		challenge	0.5643	ns	
		Interaction	0.4659	ns	
	*dscam*	EO (*M. alternifolia*)	0.1460	ns	-
		challenge	0.1796	ns	
		Interaction	0.8368	ns	
	*hsp 70*	EO (*M. alternifolia*)	0.6845	ns	-
		challenge	0.0494	*	
		Interaction	0.3857	ns	
	hmgb	EO (*M. alternifolia*)	0.7357	ns	-
		challenge	0.0471	*	
		Interaction	0.9375	ns	
	sod	EO (*M. alternifolia*)	0.6114	ns	-
		challenge	0.1680	ns	
		Interaction	0.9285	ns	
	pxn	EO (*M. alternifolia*)	0.1248	ns	-
		challenge	0.0947	ns	
		Interaction	0.0745	ns	
	tgase1	EO (*M. alternifolia*)	0.4522	ns	-
		challenge	0.1306	ns	
		Interaction	0.1762	ns	
	tgase2	EO (*M. alternifolia*)	0.1888	ns	-
		challenge	0.3352	ns	
		Interaction	0.6450	ns	
24h	*lgbp*	EO (*M. alternifolia*)	0.0035	**	-
		challenge	0.3355	ns	
		Interaction	0.1960	ns	
	*dscam*	EO (*M. alternifolia*)	0.0450	*	-
		challenge	0.5783	ns	
		Interaction	0.1452	ns	
	*hsp 70*	EO (*M. alternifolia*)	0.8309	ns	antagonistic
		challenge	0.3049	ns	
		Interaction	0.0324	*	
	hmgb	EO (*M. alternifolia*)	0.4211	ns	-
		challenge	0.0818	ns	
		Interaction	0.9547	ns	
	sod	EO (*M. alternifolia*)	0.0112	*	-
		challenge	0.3028	ns	
		Interaction	0.1879	ns	
	pxn	EO (*M. alternifolia*)	0.0444	*	-
		challenge	0.0269	*	
		Interaction	0.0866	ns	
	tgase1	EO (*M. alternifolia*)	0.0002	***	-
		challenge	0.0173	*	
		Interaction	0.1867	ns	
	tgase2	EO (*M. alternifolia*)	0.0017	**	-
		challenge	0.6760	ns	
		Interaction	0.7809	ns	

B					
Time point	Gene	Factors	*P* value	*P* value summary	Interaction
6h	*lgbp*	EO (*Litsea citrata*)	0.6656	ns	-
		challenge	0.9053	ns	
		Interaction	0.6053	ns	
	*dscam*	EO (*L. citrata*)	0.5382	ns	-
		challenge	0.2184	ns	
		Interaction	0.9115	ns	
	*hsp 70*	EO (*L. citrata*)	0.5327	ns	-
		challenge	0.1660	ns	
		Interaction	0.5136	ns	
	hmgb	EO (*L. citrata*)	0.5772	ns	-
		challenge	0.0055	**	
		Interaction	0.8219	ns	
	sod	EO (*L. citrata*)	0.2664	ns	-
		challenge	0.6359	ns	
		Interaction	0.1313	ns	
	pxn	EO (*L. citrata*)	0.0545	ns	-
		challenge	0.7649	ns	
		Interaction	0.9075	ns	
	tgase1	EO (*L. citrata*)	0.8293	ns	-
		challenge	0.2389	ns	
		Interaction	0.1047	ns	
	tgase2	EO (*L. citrata*)	0.1274	ns	-
		challenge	0.7944	ns	
		Interaction	0.1445	ns	
12h	*lgbp*	EO (*L. citrata*)	0.2981	ns	-
		challenge	0.7485	ns	
		Interaction	0.8852	ns	
	*dscam*	EO (*L. citrata*)	0.0045	**	-
		challenge	0.0230	*	
		Interaction	0.1637	ns	
	*hsp 70*	EO (*L. citrata*)	0.0945	ns	-
		challenge	0.0241	*	
		Interaction	0.1816	ns	
	hmgb	EO (*L. citrata*)	0.4481	ns	antagonistic
		challenge	0.9420	ns	
		Interaction	0.0263	*	
	sod	EO (*L. citrata*)	0.5051	ns	-
		challenge	0.8230	ns	
		Interaction	0.1217	ns	
	pxn	EO (*L. citrata*)	0.7266	ns	-
		challenge	0.0039	**	
		Interaction	0.7825	ns	
	tgase1	EO (*L. citrata*)	0.4951	ns	-
		challenge	0.1562	ns	
		Interaction	0.2472	ns	
	tgase2	EO (*L. citrata*)	0.2764	ns	-
		challenge	0.2137	ns	
		Interaction	0.4248	ns	
24h	*lgbp*	EO (*L. citrata*)	0.4771	ns	-
		challenge	0.4263	ns	
		Interaction	0.8789	ns	
	*dscam*	EO (*L. citrata*)	0.1625	ns	-
		challenge	0.3050	ns	
		Interaction	0.7909	ns	
	*hsp 70*	EO (*L. citrata*)	0.0109	*	-
		challenge	0.0077	**	
		Interaction	0.4646	ns	
	hmgb	EO (*L. citrata*)	0.2385	ns	-
		challenge	0.4116	ns	
		Interaction	0.4688	ns	
	sod	EO (*L. citrata*)	0.0014	**	-
		challenge	0.7530	ns	
		Interaction	0.6294	ns	
	pxn	EO (*L. citrata*)	0.0053	**	-
		challenge	0.1603	ns	
		Interaction	0.1004	ns	
	tgase1	EO (*L. citrata*)	0.0005	***	-
		challenge	0.0738	ns	
		Interaction	0.4440	ns	
	tgase2	EO (*L. citrata*)	0.1593	ns	-
		challenge	0.6452	ns	
		Interaction	0.5054	ns	

C					
Time point	Gene	Factors	*P* value	*P* value summary	Interaction
6h	*lgbp*	EO (*Eucalyptus citriodora*)	0.2767	ns	-
		challenge	0.6524	ns	
		Interaction	0.8772	ns	
	*dscam*	EO (*E. citriodora*)	0.0964	ns	-
		challenge	0.0066	**	
		Interaction	0.0705	ns	
	*hsp 70*	EO (*E. citriodora*)	0.7276	ns	-
		challenge	0.3050	ns	
		Interaction	0.8937	ns	
	hmgb	EO (*E. citriodora*)	0.0694	ns	antagonistic
		challenge	0.1342	ns	
		Interaction	0.0042	**	
	sod	EO (*E. citriodora*)	0.6201	ns	antagonistic
		challenge	0.3071	ns	
		Interaction	0.0130	*	
	pxn	EO (*E. citriodora*)	0.8847	ns	-
		challenge	0.1854	ns	
		Interaction	0.2631	ns	
	tgase1	EO (*E. citriodora*)	0.5771	ns	antagonistic
		challenge	0.0188	*	
		Interaction	0.0082	**	
	tgase2	EO (*E. citriodora*)	0.0052	**	antagonistic
		challenge	0.6609	ns	
		Interaction	0.0079	**	
12h	*lgbp*	EO (*E. citriodora*)	0.0504	ns	-
		challenge	0.9434	ns	
		Interaction	0.7976	ns	
	*dscam*	EO (*E. citriodora*)	0.0032	**	-
		challenge	0.1783	ns	
		Interaction	0.8676	ns	
	*hsp 70*	EO (*E. citriodora*)	0.8764	ns	-
		challenge	0.5147	ns	
		Interaction	0.6000	ns	
	hmgb	EO (*Eucalyptus citriodora*)	0.7198	ns	-
		challenge	0.0215	*	
	Interaction	0.6577	ns	
	sod	EO (*E. citriodora*)	0.8301	ns	-
		challenge	0.0914	ns	
		Interaction	0.8695	ns	
	pxn	EO (*E. citriodora*)	0.0408	*	-
		challenge	0.1499	ns	
		Interaction	0.5417	ns	
	tgase1	EO (*E. citriodora*)	0.9968	ns	-
		challenge	0.1144	ns	
		Interaction	0.2009	ns	
	tgase2	EO (*E. citriodora*)	0.7905	ns	-
		challenge	0.1167	ns	
		Interaction	0.3772	ns		-
24h	*lgbp*	EO (*E. citriodora*)	0.1281	ns	-
		challenge	0.8545	ns	
		Interaction	0.7889	ns	
	*dscam*	EO (*E. citriodora*)	0.0344	*	-
		challenge	0.0041	**	
		Interaction	0.1328	ns	
	*hsp 70*	EO (*E. citriodora*)	0.0567	ns	-
		challenge	0.0059	**	
		Interaction	0.1879	ns	
	hmgb	EO (*E. citriodora*)	0.7132	ns	antagonistic
		challenge	0.8458	ns	
		Interaction	0.0250	*	
	sod	EO (*E. citriodora*)	0.1185	ns	-
		challenge	0.4891	ns	
		Interaction	0.6296	ns	
	pxn	EO (*E. citriodora*)	0.5817	ns	antagonistic
		challenge	0.0816	ns	
		Interaction	0.0292	*	
	tgase1	EO (*E. citriodora*)	0.0001	****	synergistic
		challenge	0.0001	****	
		Interaction	0.0010	**	
	tgase2	EO (*E. citriodora*)	0.1744	ns	antagonistic
		challenge	0.0125	*	
		Interaction	0.0096	**	

The larvae were treated with either three different essential oils at an optimized concentration or challenged with/without V. campbellii at 10^7^ cells/ml. Data represent the mean of four replicates. (Two-way ANOVA; ns, no significant differences; *, P < 0.05; **, P < 0.01; ***, P < 0.001; ****, P < 0.0001; no interaction); EO, essential oil.

### EOs Regulate the *In Vitro* Virulence of Pathogenic *V. campbellii*

**Figure 5:**
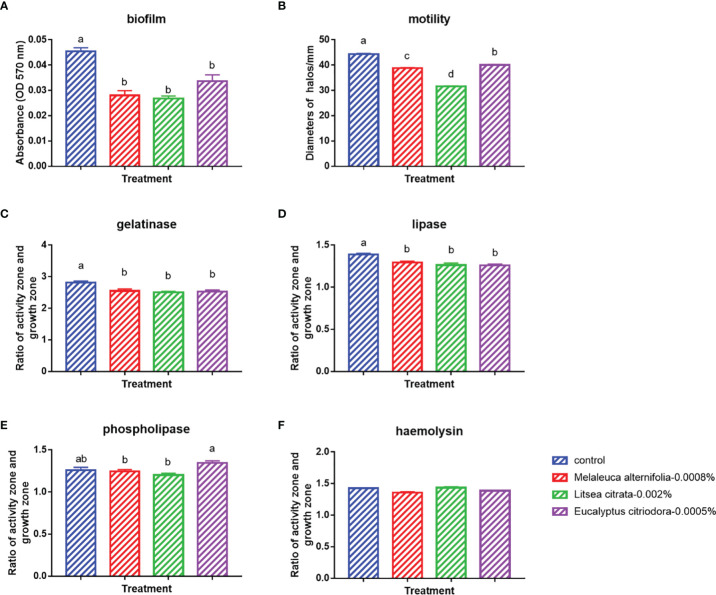
*In vitro* tests on the bacterial virulence factors of *Vibrio campbellii* treated with the selected essential oils at an optimized dose. **(A)** Biofilm formation of *V. campbellii* in marine broth. **(B)** Swimming motility of *V. campbellii* on soft LB_35_ agar after 24 h of incubation at 28°C. **(C)** Gelatinase assay of *V. campbellii* on LB_35_ agar supplemented with 0.5% gelatin after 48 h of incubation at 28°C. **(D)** Lipase assay of *V. campbellii* on LB_35_ agar supplemented with 1% Tween 80 after 2–4 days of incubation at 28°C. **(E)** Phospholipase assay of *V. campbellii* on LB_35_ agar supplemented with 1% egg yolk after 2–4 days of incubation at 28°C. **(F)** Hemolytic assay of *V. campbellii* on LB_35_ agar supplemented with 5% defibrinated sheep blood after 48 h of incubation at 28°C. Control: no EO added but consisted of 1% of DMSO. The error bars of the graphs represented standard error. Different letters indicated significant difference (*P* < 0.05).

**Figure 6:**
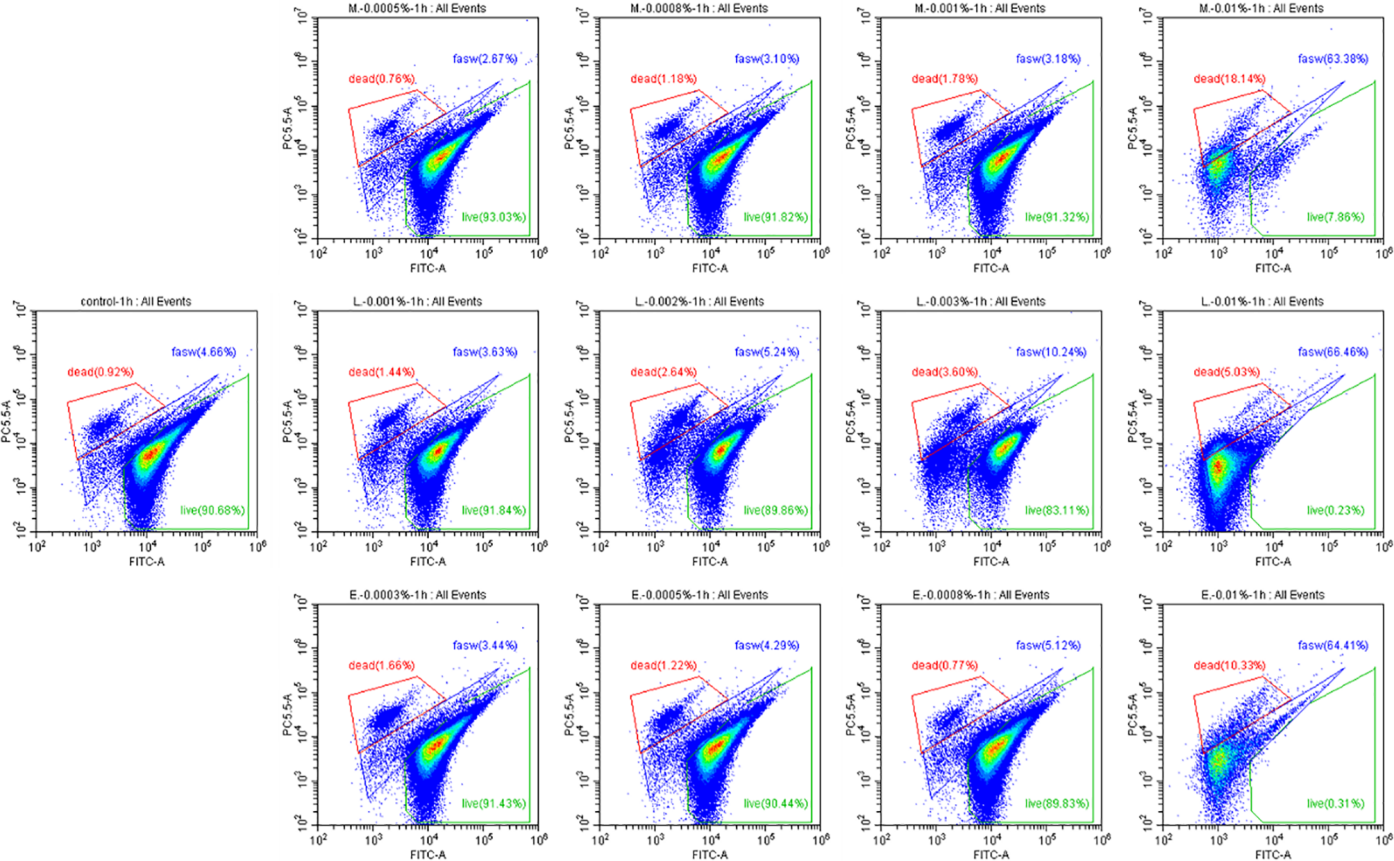
Percentage of live and dead cells of *Vibrio campbellii* with or without the addition of essential oils. The first line from left to right was the group of essential oil *Melaleuca alternifolia* at the concentrations of 0.0005, 0.0008, 0.001, and 0.01%; the second line from left to right was control group, the group of essential oil *Litsea citrata* at the concentrations of 0.001, 0.002, 0.003, and 0.01%; and the third line from left to right was the group of essential oil *Eucalyptus citriodora* at the concentrations of 0.0003, 0.0005, 0.0008, and 0.01%. Control: no EO added but consisted of 1% of DMSO. The plot of live cells was in the green frame, dead cells was in the red frame, and damage cells or other were in the blue frame.

**Figure 7:**
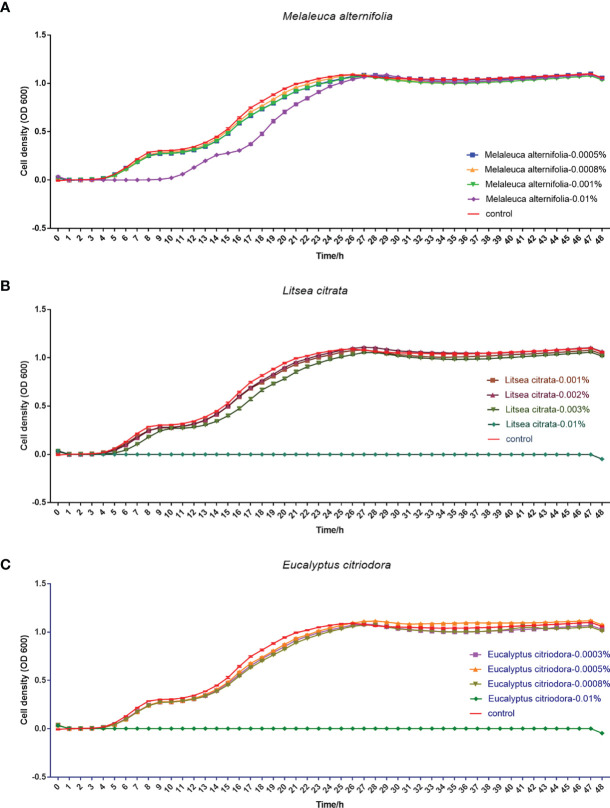
Regrowth curve of *Vibrio campbellii* after incubation with or without essential oil *Melaleuca alternifolia* **(A)**, *Litsea citrata* **(B)**, and *Eucalyptus citriodora* **(C)** at different concentrations for 1 h. The group of essential oil *M. alternifolia* at the concentrations of 0.0005, 0.0008, 0.001, and 0.01%; the group of essential oil *L. citrata* at the concentrations of 0.001, 0.002, 0.003, and 0.01%; and the group of essential oil *E. citriodora* at the concentrations of 0.0003, 0.0005, 0.0008, and 0.01%. The control group contained 1% DMSO. Error bars represented the standard error of four replicates.

Finally, the effects of EOs on the virulence factors and the cell density of *V. campbellii* were investigated. The EOs of *M. alternifolia* at 0.0008%, *L. citrata* at 0.002%, and *E. citriodora* at 0.0005% significantly decreased biofilm formation, swimming motility, gelatinase activity, and lipase activity *in vitro* (**Figure 5** and **Supplementary Information 3**). However, there was no significant effect on phospholipase or hemolytic activity. There was no significant change in the number of total cells, live cells, and dead cells detected in the *V. campbellii* supplemented with the EOs of *M. alternifolia* (0.0008%), *L. citrata* (0.002%), and *E. citriodora* (0.0005%) (**Figure 6**, **Supplementary Information 4**, and **Table 5**). No significant regrowth was observed when *V. campbellii* was treated with *L. citrata* at 0.01% and *E. citriodora* at 0.01%, while the EO of *M. alternifolia* at 0.01% delayed the bacterial exponential growth phase. There is no significant difference in regrowth performance in other analyzed concentrations compared to the control group (**Figure 7**).

**Table 5:** Average total cells count, live cells count, and dead cells count of *Vibrio campbellii* with or without essential oils at different concentrations, obtained from flow cytometer.

Group	Average total cells count (×10^3^ cell/ml)	Live cells count (×10^3^ cell/ml)	Dead cells count (×10^3^ cell/ml)
Negative control	2,907.13 ± 7.56	2,645.69 ± 13.27	38.65 ± 10.84
*Melaleuca alternifolia*—0.0005%	2,643.05 ± 10.45	2,461.45 ± 7.50	25.87 ± 3.25
*M. alternifolia*—0.0008%	2,769.7 ± 26.08	2,589.03 ± 15.96	35.07 ± 4.06
*M. alternifolia*—0.001%	2,655.66 ± 11.82	2,453.48 ± 16.98	39.54 ± 4.84
*M. alternifolia*—0.01%	229.83 ± 2.74	18.68 ± 0.11	42.99 ± 0.19
*Litsea citrata*—0.001%	2,220.74 ± 18.12	2,053.67 ± 19.97	34.53 ± 3.71
*L. citrata*—0.002%	1,944.87 ± 4.05	1,757.44 ± 5.06	56.03 ± 2.44
*L. citrata*—0.003%	1,285.62 ± 20.30	1,078.70 ± 17.78	44.25 ± 1.97
*L. citrata*—0.01%	1,654.60 ± 24.84	3.94 ± 0.05	82.41 ± 0.51
*Eucalyptus citriodora*—0.0003%	2,731.842 ± 27.60	2,497.86 ± 20.99	34.29 ± 8.81
*E. citriodora*—0.0005%	2,847.15 ± 30.46	2,565.29 ± 21.95	31.33 ± 9.17
*E. citriodora*—0.0008%	2,789.38 ± 34.46	2,536.09 ± 26.24	36.84 ± 7.60
*E. citriodora*—0.01%	190.05 ± 4.59	0.8 ± 0.02	25.36 ± 0.37

## Discussion

We observed that the EOs of *M. alternifolia* and *L. citrata* increase the survival of *Artemia* against the pathogenic *V. campbellii* BB120 strain *in vivo*. Our data implied that supplementation of EOs induced a protective response in *Artemia* when challenged with *V. campbellii*. EOs seemed to be responsible for stimulating the immune system of *Artemia in vivo*. Moreover, the EOs diminished the virulence factors production without inhibiting the growth of the pathogen *in vitro*.

The bacterial species *V. campbellii* is an important pathogen in the intensive rearing of aquatic animals, where mortality can be as high as 100% (1). The use of antibiotics and disinfectants has limited success in controlling this pathogen due to the emergence of bacterial resistance (18). Hence, an alternative approach is needed to control vibriosis. The EOs of *M. alternifolia*, *L. citrata*, and *E. citriodora* were previously observed to inhibit or decrease the growth of *V. campbellii* BB120 *in vitro*. This motivated us to investigate whether they can be applied as a disease-control agent in aquaculture. Therefore, using the axenic brine shrimp and *V. campbellii* as a host-pathogen model, the potential beneficial effect of the EOs was verified and the potential mode of action investigated.

To this end, the toxicity of each EO was firstly evaluated in brine shrimp larvae. The results showed that EOs of *M. alternifolia*, *L. citrata*, and *E. citriodora* below 0.001, 0.002, and 0.0005% concentration had no effect on the larvae survival, which indicated that *Artemia* was more tolerant to essential oil *L. citrata*, followed by *M. alternifolia* and *E. citriodora*. Hence, compared to the essential oil *M. alternifolia* and *E. citriodora*, there might be more flexibility in applying the EO of *L. citrata* in aquaculture.

Next, to evaluate the protective effect of EO in the brine shrimp, the optimized doses of each EO were applied to the standard *Artemia in vivo* challenge assay. The survival results indicated that the EOs of *M. alternifolia* at 0.0008% and *L. citrata* at 0.002% significantly improved the survival of brine shrimp challenged with *V. campbellii*, while the EO of *E. citriodora* had limited protection. To analyze this differential survival protection, it was tried to link survival with the influence of EO on the immune system of brine shrimp, especially the immune genes.

The *Artemia* group exposed to the EO *M. alternifolia* and challenged with *V. campbellii* exhibited a significant synergistic interaction in the *dscam* and *hsp 70* gene expression level at the 6 h time point, culminating for the latter gene in a more than two-fold higher expression at 24 h after *Vibrio* exposure. Previous studies showed that infected rats treated with *M. alternifolia* had increased levels of IgG (19), and treatment ameliorates the cytokine response of silver catfish during *Aeromonas hydrophila* infection (20). These studies were mainly focused on antibody and cytokine levels in serum. In the present study, significant antagonistic interaction was observed with the EO *M. alternifolia* treatment in combination with high expression of *tgase1*, indicating that *M. alternifolia* might protect *Artemia* by an enhanced immune response during the first 24 h of *Vibrio* exposure.

In the *Artemia* group treated with EO *E. citriodora*, a significant antagonistic interaction at 6 h in the expression of *hmgb*, *sod*, *tgase1*, and *tgase2* gene, and at 24 h in the *hmgb*, *pxn*, and *tgase2* gene expression was observed, culminating a two-fold higher expression of *hsp70* and *tgase1* after 24-h challenge in the presence of this EO. Yet, the *Artemia* group exposed to *E. citriodora* group only displayed a limited increase in survival after challenge. It was known that the generation of protective immunity was an energy-consuming process (21). In invertebrates, each of the effector systems involved in the immune response may carry a different cost when activated, and their relative expression may shape the cost of the whole immune response to a standard challenge (6). Although *E. citriodora* activate, *e.g., tgase1*, known to be important for protection, it might be that this EO activated other immune-related genes not measured here, resulting in a high energetic cost. However, this mechanistic explanation is speculative at this stage and should be verified by, e.g., analyzing the complete immunological response in *Artemia* over a 24-h time span.

There were no interactive effects upon EO *L. citrata* exposure and challenge in the expression of immune-related genes (but a high *tgase1* exposure after 24-h challenge). Yet, the *L. citrata* group displayed a very high survival, and a protection closed to 100%. It was interesting to observe that there was a high *sod* gene expression (more than two-fold) in the *L. citrata* group at 24 h both with and without challenge (and not observed with the other two EOs). It has been reported that numerous short-lived and highly reactive oxygen species (ROS) such as superoxide $\left({\text{O}}_{2}^{-}\right)$ , hydroxyl radical, and hydrogen peroxide were continuously generated *in vivo*, especially during an immune response (22). Oxidant/antioxidant imbalance was thought to be partially involved in the pathogenesis of the disorders (22). Maybe the EO *L. citrata* exposure helped to mount a balanced immune response in which radicals generated were neutralized by the expression of ROS-neutralizing genes such as SOD, contributing to a high survival under challenge. Summarizing, the EOs tested seemed to modulate the immune response in *Artemia*, each of them in their particular way. Only in two cases these seemed to contribute to a substantial increase in survival. It is possible that the panel of verified immune-related genes does not generate the complete picture of the immune response upon exposure, and hence an unequivocal temporal picture between immune gene expression and *Artemia* survival for these three EOs could not be generated.

In addition to the effect of EO on the immune system of *Artemia in vivo*, the impact of the EOs on the pathogenic *V. campbellii* strain was also investigated in a series of *in vitro* assays. The flow cytometer results showed that the amount of live cell and dead cell of bacteria in the essential oil of *M. alternifolia* at 0.0008%, *L. citrata* at 0.002%, and *E. citriodora* at 0.0005% were the same magnitude as that in the control group, showing *V. campbellii* were not killed by the EOs at the tested concentrations. For the regrowth curve, bacteria in the essential oil of *M. alternifolia* at 0.0008%, *L. citrata* at 0.002%, and *E. citriodora* at 0.0005% exhibited the same growth curve as that in the control group, indicating that the growth performance of *V. campbellii* was not affected by the EOs at the tested concentrations. The EOs might act as a bacteriostatic or bactericidal agent. Bacteriostatic agents prevent the growth of bacteria, suppressing cellular division, while bactericidal agents kill the bacteria (23). The bacteriostatic action has a reversible character, and the microbial cells will recover their reproductive capacity (23) when the agent is removed. In contrast, the bactericidal effect is permanent, and the microbial cells are not able to regrow (23). Combined with the result from the flow cytometer and the regrowth performance, there was no bactericidal effect in the EOs at the tested concentrations. Bacterial swimming motility played a critical role in host-microbial interactions, which was also related to biofilm formation. In our study, all EOs at optimized concentration had a significant decrease in swimming motility and biofilm formation. Similar results have been reported by Domínguez-Borbor et al. (24), who observed that EOs have a profound effect on the virulence of *Vibrio* sp. The results showed that EOs (*Organum vulgare* and *M. alternifolia*) significantly inhibited the biofilm formation in *V. harveyi*, *V. campbellii*, *V. parahaemolyticus*, and *V. vulnificus*. Additionally, the EOs from different ornamental plants have also been observed to be effective against biofilms formed by *Salmonella*, *Listeria*, *Pseudomonas*, *Staphylococcus*, and *Lactobacillus* spp (25, 26). *V. campbellii* excreted various virulence factors, such as extracellular products (gelatinase, lipase, phospholipase, hemolytic, and so on) that were involved in pathogenesis (17). The results revealed that essential oils significantly decreased the production of certain virulence factors (gelatinase, lipase), whereas they had no significant effect on phospholipase or hemolytic activity. This indicated that the inhibition of swimming motility, biofilm formation, and virulence factors was to a certain degree consistent with a bacteriostatic action of the EOs at the tested concentrations.

## Conclusion

In conclusion, the results presented in this paper showed that essential oils of *M. alternifolia* at 0.0008% and *L. citrata* at 0.002% can significantly improve the survival of brine shrimp larvae when challenged with pathogenic *V. campbellii*. Our results indicated that supplementation of these two EOs enhanced immune gene expression (each of them in their particular way), possibly contributing to protective immunity in brine shrimp larvae against *V. campbellii*. Furthermore, the EOs-regulated expression of bacterial virulence factors, including decreased swimming motility and biofilm formation, might contribute in part to the protection of the brine shrimp larvae against pathogenic *V. campbellii*. However, further studies are needed to investigate the underlying protective mechanism of EOs, e.g., by analyzing the expression of a larger panel of immune-related genes. Taken together, the essential oils can be part of disease intervention strategies either based on their immunostimulatory properties or modulation of virulence factor production.

## Data Availability Statement

The original contributions presented in the study are included in the article/**Supplementary Material**. Further inquiries can be directed to the corresponding authors.

## Author Contributions

XZ: conceptualization, data curation, investigation, visualization, writing—original draft and writing—review and editing. BH: methodology and writing—review and editing. VK: methodology and writing—review and editing. AF: resources, methodology, and writing—review and editing. PD: resources, supervision, and writing—review and editing. PB: conceptualization, supervision, funding acquisition, and writing—review and editing.

## Funding

This research was supported by the China Scholarship Council (CSC 201708440251) and the Special Research Fund of Ghent University (BOF-UGent 01SC7918).

## Conflict of Interest

The authors declare that the research was conducted in the absence of any commercial or financial relationships that could be construed as a potential conflict of interest.

## Publisher’s Note

All claims expressed in this article are solely those of the authors and do not necessarily represent those of their affiliated organizations, or those of the publisher, the editors and the reviewers. Any product that may be evaluated in this article, or claim that may be made by its manufacturer, is not guaranteed or endorsed by the publisher.
