# Supplementary material for: Essential Oils Improve the Survival of Gnotobiotic Brine Shrimp (Artemia franciscana) Challenged With Vibrio campbellii
Source: Front Immunol. 2021 Oct 20;12:693932. doi: 10.3389/fimmu.2021.693932 (PMC8564362; doi:10.3389/fimmu.2021.693932)
Supplement: Supplementary file 1 [file Image_1.pdf]

Supplementary information 3:

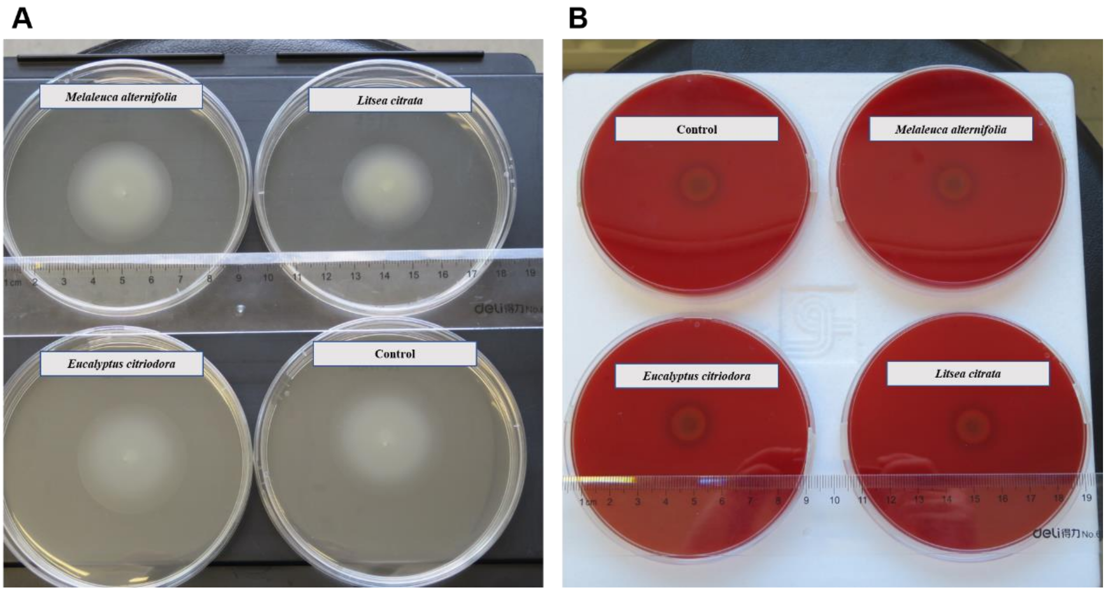

Effect of *Melaleuca alternifolia* (0.0008%), *Litsea citrata* (0.002%) and *Eucalyptus citriodora* (0.0005%) on swimming motility of *V. campbellii* on soft LB_{35} agar after 24 h of incubation at 28 ℃ (A) and hemolytic assay of *V. campbellii* on LB_{35} agar supplemented with 5% defibrinated sheep blood after 48h of incubation at 28 ℃ (B). Control: no EO added but consisted of 1% of DMSO.
