# Supplementary material for: Essential Oils Improve the Survival of Gnotobiotic Brine Shrimp (Artemia franciscana) Challenged With Vibrio campbellii
Source: Front Immunol. 2021 Oct 20;12:693932. doi: 10.3389/fimmu.2021.693932 (PMC8564362; doi:10.3389/fimmu.2021.693932)
Supplement: Supplementary file 2 [file Image_2.pdf]

Supplementary information 4:

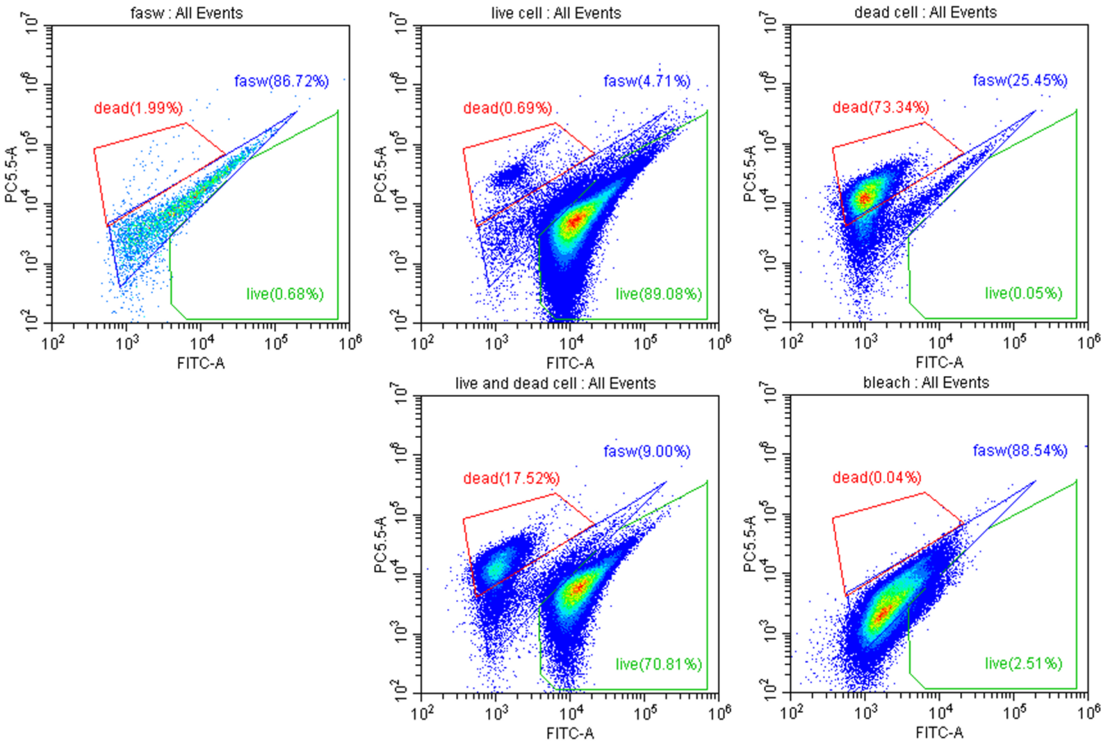

Percentage of live and dead cells of *V. campbellii* obtained from flow cytometry staining with Thiazole Orange and Propidium Iodide. The first line from left to right was FASW sample, live cells sample and dead cells sample treated with 95 °C hot water bath for 20 mins and frozen in -80 °C for 10 mins, the second line from left to right was mixture of 50% of the live cells and 50% of the dead cells sample, the dead cells treated with bleach for 10 mins. The Plot of live cells was in the green frame, dead cells was in the red frame, and damage cell or other was in the blue frame.
