# Supplementary material for: Essential Oils Improve the Survival of Gnotobiotic Brine Shrimp (Artemia franciscana) Challenged With Vibrio campbellii
Source: Front Immunol. 2021 Oct 20;12:693932. doi: 10.3389/fimmu.2021.693932 (PMC8564362; doi:10.3389/fimmu.2021.693932)
Supplement: Supplementary file 3 [file Table_1.docx]

**Supplementary information 1：**

Three kinds of EOs used in this study with their components (≥10%) and assigned chemical class of components.

| EO of plant | Major components (≥10% v/v) | % (v/v) | Chemical class |
| --- | --- | --- | --- |
| *Melaleuca alternifolia* | TERPINEN-4-OL | 41.35 | monoterpenols |
|  | γ-TERPINENE | 20.64 | monoterpenes |
| *Litsea citrata* | CITRAL | 71.35 | aldehydes |
|  | LIMONENE | 11.53 | monoterpenes |
| *Eucalyptus citriodora* | CITRONELLAL | 80.02 | aldehydes |
