# Supplementary material for: Essential Oils Improve the Survival of Gnotobiotic Brine Shrimp (Artemia franciscana) Challenged With Vibrio campbellii
Source: Front Immunol. 2021 Oct 20;12:693932. doi: 10.3389/fimmu.2021.693932 (PMC8564362; doi:10.3389/fimmu.2021.693932)
Supplement: Supplementary file 4 [file Table_2.docx]

**Supplementary information 2:**

The main function of the eight selected genes or their possible relationship with immunity in crustaceans.

| Gene | Function or their relationship with immunity | Reference |
| --- | --- | --- |
| *lgbp* | Pattern recognition proteins (PRPs), involved to induce encapsulation, phagocytosis, nodule formation, clotting, synthesis of antimicrobial peptides and activate prophenoloxidase (ProPO) system | (1) |
| *dscam* | Produce isoforms can bind different bacteria depending on exon composition, alternative exons change upon infection, increasing phagocytosis rates | (2) |
| *hsp 70* | Folding polypeptides, repair of partially denatured proteins and inhibit protein aggression; shielding cells against injury due to pathogens, activating Toll-like receptors, delivering inflammatory signals and stabilizing key proteins involved in pathogen destruction | (3) |
| *hmgb* | Stabilize nucleosome formation, acted as transcription- factor that regulates gene expressions; induced cytokines and inflammatory responses | (4) |
| *sod* | Antioxidant defense; dismutase superoxide radicals into hydrogen peroxide | (5) |
| *pxn* | A cell adhesive protein associated with ProPo system | (6) |
| *tagse* | Involved in the blood coagulation system of many crustaceans | (7) |

1. Cheng W, Liu C-H, Tsai C-H, Chen J-C. Molecular Cloning and Characterisation of a Pattern Recognition Molecule, Lipopolysaccharide- and b-1, 3-Glucan Binding Protein (LGBP) From the White Shrimp *Litopenaeus vannamei*. Fish Shellfish Immunol (2005) 18(4):297–310. doi: 10.1016/j.fsi.2004.08.002

2. Dong Y, Taylor HE, Dimopoulos G. AgDscam, a Hypervariable Immunoglobulin Domain-Containing Receptor of the *Anopheles Gambiae* Innate Immune System. PloS Biol (2006) 4(7) e229. doi: 10.1371/ journal.pbio.0040229

3. Roberts RJ, Agius C, Saliba C, Bossier P, Sung YY. Heat Shock Proteins (Chaperones) in Fish and Shellfish and Their Potential Role in Relation to Fish Health: A Review. J Fish Dis (2010) 33(10):789–801. doi: 10.1111/j.1365- 2761.2010.01183.x

4. Tang D, Kang R, Zeh Iii HJ, Lotze MT. High-Mobility Group Box 1, Oxidative Stress, and Disease. Antioxid Redox Signal (2011) 14(7):1315–35. doi: 10.1089/ ars.2010.3356

5. Rahal A, Kumar A, Singh V, Yadav B, Tiwari R, Chakraborty S, et al. Oxidative Stress, Prooxidants, and Antioxidants: The Interplay. BioMed Res Int (2014) 2014:1-19. doi: 10.1155/2014/761264

6. Sritunyalucksana K, Wongsuebsantati K, Johansson MW, Söderhäll K. Peroxinectin, a Cell Adhesive Protein Associated With the proPO System From the Black Tiger Shrimp, *Penaeus Monodon*. Dev Comp Immunol (2001) 25(5-6):353–63. doi: 10.1016/S0145-305X(01)00009-X

7. Maningas MBB, Kondo H, Hirono I, Saito-Taki T, Aoki T. Essential Function of Transglutaminase and Clotting Protein in Shrimp Immunity. Mol Immunol (2008) 45(5):1269–75. doi: 10.1016/j.molimm.2007.09.016
